# Quality of Life of Primary Caregivers of Children With Cerebral Palsy From a Family Perspective

**DOI:** 10.7759/cureus.49378

**Published:** 2023-11-25

**Authors:** Kadi A Alhumaidi, Meznah O Alshwameen, Maram S Alsayed, Dana K Alqoaer, Reema S Albalawi, Sarah M Alanzi, Amjad F Alharthe, Hind Abdulaziz Subayyil Alanazi

**Affiliations:** 1 Medicine, Unaizah College of Medicine, Qassim University, Unaizah, SAU; 2 Pediatrics, Maternity and Children Hospital, Ministry of Health, Tabuk, SAU; 3 Medicine, University of Tabuk, Tabuk, SAU; 4 Medicine and Surgery, University of Tabuk, Tabuk, SAU; 5 Internal Medicine, Faculty of Medicine, University of Tabuk, Tabuk, SAU

**Keywords:** saudi arabia, tabuk, cerebral palsy, children, caregivers, quality of life

## Abstract

Background: Cerebral palsy is a heterogeneous group of permanent non-progressive disorders affecting the development of movement and posture, varying in severity, interfering with daily activity, and associated with multiple *comorbidities*. Previous studies in different regions of Saudi Arabia have shown links between caregivers’ mental health and children’s well-being. However, the lack of such research in the Tabuk region necessitates the development of a new survey to assess caregivers’ quality of life in this specific area.

Methodology: This was a cross-sectional study, conducted during 2022-2023. It included the caregivers of Saudi children with cerebral palsy in the Tabuk region. Data was collected using an online questionnaire and was analyzed using the SPSS program (IBM Corp., Armonk, NY).

Results: The study included 63 participants. Mothers (50.8%) and fathers (46.0%) were the primary caregivers. The comorbidities of cerebral palsy children like seizures (28.6%) and learning difficulties (19.0%) were prevalent for cerebral palsy children. Around 20.6% of participants received home health care. In terms of family quality of life, the majority expressed satisfaction with family interaction, parenting, and emotional well-being, although mixed feelings were observed.

Conclusion: The majority of caregivers reported satisfaction in various domains of family quality of life, particularly in family interaction and parenting. However, a notable percentage expressed dissatisfaction in some areas, highlighting the complex emotional and practical challenges faced by these caregivers.

## Introduction

Cerebral palsy (CP) refers to a set of non-progressive, but often changing motor impairment syndromes secondary to anomalies or lesions of the brain appearing before or after birth. The estimated CP prevalence of 1.6 per 1,000 lives and the incidence of CP was 0.41% [1,2]. Causes include Low birth weight, prematurity, congenital malformations, and kernicterus. When children fail to meet motor milestones or exhibit anomalies such as asymmetrical gross motor function, hypertonia, or hypotonia, they are often diagnosed with CP during the first 18 months of life except for the mildest kinds. The body areas that are affected and explanations of the most common type of motor impairment might help further define CP. CP comorbidities include epilepsy, learning disabilities, behavioral issues, and sensory impairment. Many of these children suffer from a single motor defect, intellectual giftedness may occur in some affected children. Parents' physical health and social well-being were found to be negatively impacted by the responsibility of raising a child with CP, particularly mothers who, in comparison to moms of healthy children, are more likely to experience stress and sadness [3].

A cross-sectional study in Taif City was conducted among 232 moms of CP children between the ages of five and 18 to assess mothers' depression and anxiety, as well as children's quality of life, it was found that 55.5% of moms showed varying levels of depression, while 47.4% and 21.6% experienced moderate to severe anxiety, respectively [4].

Moreover, a cross-sectional study was carried out among carers of children with CP by using the Depression Anxiety Stress Scale-21, in National Guard Health Affairs-Jeddah, Saudi Arabia total of 40 caregivers made up the study sample, and 72.5% of them were mothers. 12.5% (n = 5) of the carers had moderate depression scores on the Depression Anxiety Stress Scale-21, 10% (n = 4) had extremely severe depression, and 10% (n = 4) had significant anxiety. Additionally, among the carers, 12.5% (n = 5), 15% (n = 6), and 7.5% (n = 3) reported having moderate, severe, and extremely severe stress levels, respectively. Significant (p = 0.05). The relationship was found between caregivers' depression, anxiety, and stress ratings and the influence on their dependent children's vision, frequent hospital admissions, and frequent trips to the emergency room. Higher caregiver anxiety scores were also substantially associated with an increase in pediatric intensive care unit admissions over the previous year [5].

While we were searching about this topic, we noticed insufficient studies assessing the quality of life of primary caregivers of children with CP besides the role of home health care in Saudi Arabia in improving the quality of life for them. In our study, we aim to assess it by developing a survey that targets the caregivers of children with CP. According to our knowledge, there is no previous study to assess this matter in Tabuk region, Saudi Arabia.

## Materials and methods

This cross-sectional study was conducted in the Tabuk region of Saudi Arabia from December 2022 to October 2023 through a self-administered questionnaire that was distributed in the Maternity and Children's Hospital to the primary caregivers of children with CP.

Both descriptive and inferential statistical analysis of the data was carried out. Simple descriptive statistics of the sociodemographic characteristics and other categorical variables in the form of frequencies and percentages were calculated and tabulated. In the write-up, the percentages for very dissatisfied/dissatisfied categories and very satisfied/satisfied categories were combined for easier interpretation. For continuous variables means, medians and IQRs (Inter-Quartile Ranges) were reported as measures of central tendency and dispersion respectively owing to the relatively non-normal distribution of variables assessed by the Shapiro-Wilk Test (p<0.001).

For the 22 questions assessing the family quality of life, scoring was done as per established criteria. The scores assigned were strongly disagree = 1, disagree = 2, neutral =3, agree = 4, strongly agree = 5. The scores were summed up for each of the 5 domains within the family quality of life scale i.e., family interaction, parenting, emotional well-being, physical/material well-being, and disability-related support, and for the total scale as a whole. The total family quality of life scores was compared among participants of different sociodemographic characteristics. The comparison involved inferential statistical analysis namely the non-parametric Mann-Whitney U test and the Kruskal-Wallis test. Significance was established at a p-value of 0.05 indicating a 95% confidence interval. All statistical calculations were performed using IBM SPSS version 27.0.1 (IBM Corp., Armonk, NY).

Ethical approval was sought from the Tabuk Institutional Review Board, Saudi Arabia, and participants were ensured confidentiality and the freedom to withdraw from the study at any time. Informed consent was taken before filling out the questionnaire.

## Results

Sociodemographic characteristics

The sample consisted of 63 participants, primarily aged between 30 and 44 years (68.3%), with smaller percentages in the 15-29 years age group (20.6%) and the 45-65 years age group (11.1%). The majority of participants were married (81.0%), with a substantial portion reporting family income of less than 10,000 Saudi Riyal (68.3%), followed by incomes ranging from 10,000 to 15,000 Saudi Riyal (20.6%), and more than 15,000 Saudi Riyal (11.1%). In terms of educational levels, secondary education was the most prevalent (41.3%), followed by a bachelor's degree (34.9%). A smaller percentage were illiterate (12.7%), and some had completed middle (7.9%) or primary (3.2%) education. When considering the kinship relationship with the child, the majority of participants were mothers (50.8%), while fathers represented a significant portion (46.0%). Only a few reported being sisters or brothers of the children. The hours spent with the child varied, with 42.9% dedicating more than 18 hours, 31.7% spending from six to 12 hours, and 25.4% spending less than six hours. Some children had comorbidities, with seizures (28.6%) and learning/cognitive difficulties (19.0%) being the most common, followed by vision problems (12.7%) and constipation (11.1%). About 20.6% of the participants were enrolled in home health care, while the majority (79.4%) were not. This sociodemographic information provides a valuable context for understanding the caregivers in this study and their potential influence on the quality of life of children with CP (Table 1).

**Table 1:** Distribution of the sample according to biopathographic variables. ​

		N	%
Age (years)	30-44	43	68.3%
	15-29	13	20.6%
	45-65	7	11.1%
Marital Status	Married	51	81.0%
	Divorced/Widow	12	19.0%
Family Income	Less than 10,000 Saudi Riyal	43	68.3%
	From 10,000 to 15,000 Saudi Riyal	13	20.6%
	More than 15,000 Saudi Riyal	7	11.1%
Educational level	Secondary	26	41.3%
	Bachelor's	22	34.9%
	Illiterate	8	12.7%
	Middle	5	7.9%
	Primary	2	3.2%
Kinship relationship with the child	Mother	32	50.8%
	Father	29	46.0%
	Sister	1	1.6%
	Brother	1	1.6%
Hours spent with the child	More than 18 hours	27	42.9%
	From 6 to 12 hours	20	31.7%
	Less than 6 hours	16	25.4%
Other diseases associated with the child	Seizures	18	28.6%
	Learning and cognitive difficulties	12	19.0%
	Vision problems	8	12.7%
	Constipation	7	11.1%
	Swallowing problems	5	7.9%
	None	5	7.9%
	Difficulties in communication	4	6.3%
	Behavioral problems	4	6.3%
Enrolled in home health care	No	50	79.4%
	Yes	13	20.6%

Family quality of life responses

Family Interaction

For the family interaction sub-domain, regarding the enjoyment of spending time together, the majority of participants (79.4%) expressed satisfaction, with 39.7% indicating they were very satisfied. In contrast, 11.1% showed dissatisfaction with various degrees of intensity (4.8% very dissatisfied and 6.3% dissatisfied). Similarly, for family members talking frankly to each other (73.0% satisfied, 14.3% dissatisfied) and solving problems together (72.5% satisfied, 14.3% dissatisfied), the majority reported satisfaction, with a small percentage expressing dissatisfaction.

Parenting

In the parenting sub-domain, family members helping the child learn independence garnered satisfaction from the majority (67.7%), with 30.2% reporting they were very satisfied. A smaller proportion (19.0%) showed some level of dissatisfaction. Similar trends were observed in responses related to other aspects of parenting, with the majority reporting satisfaction, although a minority expressed varying degrees of dissatisfaction (Table 2).

**Table 2:** Responses of the participants to family interaction and parenting aspect of the family quality of life scale

	Very dissatisfied		Dissatisfied		Neutral		Satisfied		Very satisfied	
	N	%	N	%	N	%	N	%	N	%
Family Interaction										
My family enjoys spending time together	3	4.8%	4	6.3%	6	9.5%	25	39.7%	25	39.7%
My family members talk frankly to each other	1	1.6%	8	12.7%	8	12.7%	24	38.1%	22	34.9%
My family solves problems together	2	3.2%	7	11.1%	9	14.3%	28	44.4%	17	27.0%
My family members support each other	2	3.2%	4	6.3%	6	9.5%	32	50.8%	19	30.2%
My family loves each other and shows their care for each other	5	7.9%	3	4.8%	5	7.9%	30	47.6%	20	31.7%
My family is able to handle life's ups and downs	2	3.2%	6	9.5%	8	12.7%	32	50.8%	15	23.8%
Parenting										
My family members help the child learn to rely on himself	6	9.5%	6	9.5%	9	14.3%	23	36.5%	19	30.2%
My family members help the child with school work and activities	4	6.3%	5	7.9%	19	30.2%	20	31.7%	15	23.8%
My family members teach the child how to get along with others	3	4.8%	6	9.5%	12	19.0%	26	41.3%	16	25.4%
The adults in our family teach the child to make decisions	5	7.9%	6	9.5%	11	17.5%	25	39.7%	16	25.4%
The adults in my family have enough time to take care of each child's individual needs	5	7.9%	10	15.9%	10	15.9%	23	36.5%	15	23.8%

Emotional Well-Being

In the emotional well-being sub-domain, participants' responses indicated a mixture of satisfaction and dissatisfaction. The majority reported satisfaction in terms of having the support needed to relieve stress (74.6% satisfied, 11.1% dissatisfied), while emotional support from friends or others received mixed responses, with 52.4% indicating satisfaction and 30.2% dissatisfaction. Having personal free time also showed mixed feelings, with 58.7% satisfied and 17.4% dissatisfied. Similar trends were observed in terms of having help to take care of the child's special needs, with 52.4% satisfied and 27.0% dissatisfied (Table 3).

**Table 3:** Level of family satisfaction according to their interaction and parenting

	Very dissatisfied		Dissatisfied		Neutral		Satisfied		Very satisfied	
	N	%	N	%	N	%	N	%	N	%
Emotional Well-Being										
My family has the support we need to relieve stress	3	4.8%	4	6.3%	9	14.3%	30	47.6%	17	27.0%
My family members have friends or others who provide support	8	12.7%	11	17.5%	11	17.5%	22	34.9%	11	17.5%
My family members have their own free time	6	9.5%	5	7.9%	15	23.8%	24	38.1%	13	20.6%
My family has help to take care of the child's special needs	10	15.9%	7	11.1%	13	20.6%	17	27.0%	16	25.4%
Physical / Material Well-being										
My family members have transportation to get to the places they need	3	4.8%	9	14.3%	4	6.3%	28	44.4%	19	30.2%
My family gets dental care when needed	6	9.5%	11	17.5%	12	19.0%	24	38.1%	10	15.9%
My family receives the necessary medical care	8	12.7%	9	14.3%	6	9.5%	23	36.5%	17	27.0%
Disability-Related Support										
My family members with disabilities receive support to achieve their goals at school, work or home	5	7.9%	8	12.7%	9	14.3%	24	38.1%	17	27.0%
My family members with disabilities are able to form friendships	7	11.1%	10	15.9%	13	20.6%	22	34.9%	11	17.5%
My family has good relationships with care providers for individuals with disabilities	7	11.1%	5	7.9%	7	11.1%	28	44.4%	16	25.4%

Physical/Material Well-Being

Regarding physical/material well-being, the majority of participants expressed satisfaction with having transportation to needed places (74.6% satisfied, 19.1% dissatisfied) and receiving necessary medical care (63.5% satisfied, 27.0% dissatisfied). However, responses regarding receiving dental care when needed were mixed, with 54.0% satisfied and 27.0% dissatisfied (Table 3).

Disability-Related Support

In the disability-related support sub-domain, satisfaction was predominant. The majority reported satisfaction in terms of family members with disabilities receiving support to achieve their goals at school, work, or home (65.1% satisfied, 20.6% dissatisfied) and being able to form friendships (52.4% satisfied, 27.0% dissatisfied). Good relationships with care providers for individuals with disabilities were also reported, with 69.8% satisfied and 19.0% dissatisfied (Table 3).

Scores For Each Sub-Domain of FQLS

Table 4 presents the scores of participants across the various domains of the family quality of life scale. In the family interaction domain, the mean score was 23.48 (SD=5.51), with a median of 24.00 and an interquartile range (IQR) of 20.00 to 28.00. The parenting domain had a mean score of 18.17 (SD=4.71), a median of 20.00, and an IQR of 15.00 to 22.00. For emotional well-being, the mean score was 14.00 (SD=3.74), with a median of 14.00 and an IQR of 11.00 to 16.00. In the physical/material well-being domain, the mean score was 14.65 (SD=3.24), with a median of 15.00 and an IQR of 12.00 to 17.00. In the disability-related support domain, the mean score was 10.60 (SD=3.27), with a median of 12.00 and an IQR of 9.00 to 12.00. The total family quality of life score had a mean of 80.90 (SD=17.08), a median of 82.00, and an IQR of 74.00 to 89.00.

**Table 4:** Scores of the participants in each domain of the family quality of life scale

	Mean	SD	Median	IQR
Family Interaction (Scoring: 6-30)	23.48	5.51	24.00	20.00-28.00
Parenting (Scoring: 5-25)	18.17	4.71	20.00	15.00-22.00
Emotional Well-being (Scoring: 4-20)	14.00	3.74	14.00	11.00-16.00
Physical / Material Well-being (Scoring: 4-20)	14.65	3.24	15.00	12.00-17.00
Disability-Related Support (Scoring: 3-15)	10.60	3.27	12.00	9.00-12.00
Total Family Quality of Life Score (Scoring: 22-110)	80.90	17.08	82.00	74.00-89.00

## Discussion

Our study shed light on various aspects of family quality of life for caregivers of children with CP. The results indicate a high level of satisfaction among caregivers regarding family interaction. The majority of participants expressed satisfaction, particularly in terms of enjoying spending time together. This positive finding suggests that despite the challenges posed by caring for children with CP, families in Tabuk generally maintain strong bonds and find joy in their interactions. These results align with previous studies, which have emphasized the resilience of families in the face of such caregiving responsibilities in Zambia and South Africa [6,7].

In the parenting sub-domain, caregivers reported high levels of satisfaction in helping children with CP and learning independence. This is a noteworthy result, as it underscores the commitment of caregivers to support a child’s development. The satisfaction levels in parenting aspects also resonate with the findings of Dlamini et al., which highlighted the positive impact of familial support on the child’s well-being [8].

Emotional well-being is a crucial aspect of caregivers’ quality of life. The study reveals a mixed response, with satisfaction in some areas and dissatisfaction in others. For instance, caregivers reported satisfaction with the support needed to relieve stress, which is critical for their emotional well-being. These findings are in line with studies by Wang et al., which emphasized the importance of emotional support in maintaining the well-being of caregivers [9].

Physical and material well-being is another essential dimension of caregivers’ lives. The majority expressed satisfaction with having access to transportation and necessary medical care [10]. However, the mixed response regarding dental care when needed indicates an area for potential improvement. These findings are consistent with previous research by Prabhu et al., which highlighted disparities in dental care access among caregivers of children with disabilities [11].

In the domain of disability-related support, the study findings indicate a predominance of satisfaction. This is particularly encouraging as it relates to caregivers’ access to support for individuals with disabilities to achieve their goals and form friendships. These results align with research by Glinac et al., which emphasized the positive impact of supportive care providers and services in enhancing the quality of life for families of children with CP [12].

This research also assessed the family quality of life across different domains. Notably, the highest mean score was observed in the family interaction domain, suggesting that caregivers generally find satisfaction and enjoyment in their family interactions.

In contrast, the disability-related support domain had the lowest mean score, indicating potential areas where additional support and interventions may be needed. These findings are consistent with prior research, which also highlighted the importance of family interactions and support for caregivers of children with CP [13].

Lastly, study examined the influence of various sociodemographic factors on the total family quality of life score. Among the factors assessed, the number of hours spent with the child emerged as a significant determinant. Participants who spent between six and 12 hours with their child reported a significantly higher family quality of life score compared to those who spent more or less time with their child. This finding underscores the critical role of caregiving time in influencing family quality of life. This result aligns with prior studies, which emphasized the importance of time and support in the context of caregiving [14,15].

Conversely, the study did not find significant associations between the total family quality of life score and other sociodemographic characteristics, such as age, marital status, family income, educational level, kinship relationship with the child, and the presence of various child health issues. This suggests that, in this context, family quality of life is primarily influenced by the amount of time spent caring for the child [16].

It Is essential to acknowledge the limitations of this study. The sample size was relatively small, with 63 participants, which may limit the generalizability of the findings to a larger population. The study was also conducted in a specific geographic region, and the results might not be fully representative of the diversity within Saudi Arabia. Additionally, the study relied on self-reported measures, which could introduce response bias. Furthermore, the cross-sectional design of the research makes it challenging to establish causal relationships between sociodemographic factors and family quality of life. Longitudinal studies would provide a more comprehensive understanding of the dynamic nature of caregiver experiences over time. 

## Conclusions

Family interactions, parenting, emotional well-being, physical/material well-being, and disability-related support all play crucial roles in the well-being of these caregivers. Study findings underscore the need for tailored interventions to support caregivers and improve their quality of life, focusing on aspects like emotional well-being, access to healthcare, and disability-related support. Policymakers and healthcare providers should consider these findings to develop targeted support systems that enhance the well-being of caregivers, ultimately benefiting children with CP in Tabuk and similar regions.
